# Interleukin-6, -8, and TGF-β Secreted from Mesenchymal Stem Cells Show Functional Role in Reduction of Telomerase Activity of Leukemia Cell Via Wnt5a/β-Catenin and P53 Pathways

**DOI:** 10.34172/apb.2020.037

**Published:** 2020-02-18

**Authors:** Ezzatollah Fathi, Behnaz Valipour, Zohreh Sanaat, Hojjatollah Nozad Charoudeh, Raheleh Farahzadi

**Affiliations:** ^1^Department of Clinical Sciences, Faculty of Veterinary Medicine, University of Tabriz, Tabriz, Iran.; ^2^Stem Cell Research Center, Tabriz University of Medical Sciences, Tabriz, Iran.; ^3^Hematology and Oncology Research Center, Tabriz University of Medical Sciences, Tabriz, Iran.; ^4^Drug Applied Research Center, Tabriz University of Medical Sciences, Tabriz, Iran.

**Keywords:** Mesenchymal stem cells, Telomere length, Telomerase activity, Cytokines, Wnt5a/β-catenin, P53 signaling pathways

## Abstract

***Purpose:*** The effect of mesenchymal stem cells (MSCs) on the immortality features of malignant cells, such as hematologic cancerous cells, are controversial, and the associated mechanisms are yet to be well understood. The aim of the present study was to investigate the in vitro effect of bone marrow-derived MSCs (BMSCs) on the chronic myeloid leukemia cell line K562 through telomere length measurements, telomerase activity assessments, and *hTERT* gene expression. The possible signaling pathways involved in this process, including Wnt-5a/β-catenin and P53, were also evaluated.

***Methods:*** Two cell populations (BMSCs and K562 cell line) were co-cultured on transwell plates for 7 days. Next, K562 cells were collected and subjected to quantitative real-time PCR, PCR-ELISA TRAP assay, and the ELISA sandwich technique for telomere length, *hTERT* gene expression, telomerase activity assay, and cytokine measurement, respectively. Also, the involvement of the mentioned signaling pathways in this process was reported by real-time PCR and Western blotting through gene and protein expression, respectively.

***Results:*** The results showed that BMSCs caused significant decreases in telomere length, telomerase activity, and the mRNA level of *hTERT* as a regulator of telomerase activity. The significant presence of interleukin (IL)-6, IL-8, and transforming growth factor beta (TGF-β) was obvious in the co-cultured media. Also, BMSCs significantly decreased and increased the gene and protein expression of β-catenin and P53, respectively.

***Conclusion:*** It was concluded that the mentioned effects of IL-6, IL-8, and TGF-β cytokines secreted from MSCs on K562 cells as therapeutic agents were applied by Wnt-5a/β-catenin and P53 pathways

## Introduction

Chronic myeloid leukemia (CML) is a hematopoietic stem cell disorder caused by a reciprocal translocation between chromosomes 9 and 22, resulting in the BCR-ABL fusion gene.

The BCR-ABL active tyrosine kinase promotes proliferation and cell survival through several intracellular pathways, eventually leading to malignant transformation.^1^ There are different therapy options for different kinds of leukemia, and the transplantation of stem cells is one of the most important options.^2,3^ Mesenchymal stem cells (MSCs) are multipotent cells that can be differentiated into adipocytes, osteocytes, and chondrocytes and have been harvested from adult tissues including bone marrow, adipose, heart, brain, amniotic fluid, etc.^4^ According to previous research, MSCs derived from various tissues have the same characteristics, such as the same morphology, self-renewal, and multi-lineage differentiation potential, and the ability to express different cell surface markers.^5^ Given the characteristics of MSCs, they have numerous applications in cell therapy as well as regenerative medicine.^6^ Because of their ability to migrate, secrete cytokines, and kill tumors, they have received more attention in the last decade.^7^ All these properties have led to the application of MSCs as clinical agents in therapeutic strategies.^8,9^ In addition, these cells have been genetically engineered and used as strong therapeutic agents in *in vitro* experimental models.^10^

Despite some reports that MSCs inhibit tumor growth and proliferation, others suggest that MSCs accelerate tumor progression. For example, Sun et al reported that bone marrow-derived MSCs (BMSCs) promote tumor growth and improve microanatomy sites of melanoma cells.^11^ Zhang and Zhang, however, showed that BMSCs inhibited the cell proliferation of CML cells.^12^ Telomeres with TTAGGG repeats at the end of eukaryotic chromosomes are structures that protect chromosomes from genome instability and degradation.^13^ These nucleoprotein sequences are maintained by the ribonucleoprotein enzyme telomerase reverse transcriptase. In most somatic and stem cells, due to the end-replication problem, telomeres gradually shortened. In some cancers, telomerase is activated to maintain telomere length; in some others telomere length is elongated under the mechanisms called alternative lengthening of telomeres.^14^ Therefore, reducing telomerase activity and telomere length can be used as therapeutic approaches to overcome cancer. Previous studies have shown dramatically reduced telomere lengths of leukemic cells as opposed to non-leukemic T-cells in peripheral blood cells of CML patients. Furthermore, a correlation of age-adapted telomere length with disease stage and response to treatment has also been revealed.^15,16^ With all the studies that have been done about the antitumor properties of BMSCs, the precise cellular and molecular mechanisms involved in their impact on tumor progression through the study of telomere length and telomerase activity is yet to be reported. Thus, the current study reports the *in vitro* effects of BMSCs on the mortality of the CML cell line by investigating the telomere length, telomerase activity, and gene expression of telomerase components. The possible signaling pathways involved in this process including Wnt-5a/β-catenin and P53 were also evaluated

## Materials and Methods

### Isolation of rat BMSCs

**Figure 1:**
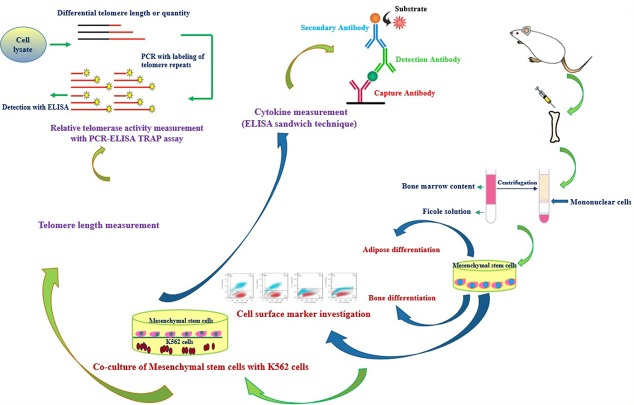
An overview of the experimental procedures that have been done in this paper.

BMSCs was isolated as described previously by Blanc et al and Fathi et al.^17,18^ In brief, after giving ethical consent, 5 (5- to 8-week-old) rats were euthanized with an overdose of ketamine/xylazine and bone marrow contains was flushed with phosphate-buffered saline (PBS) supplemented with 5% fetal bovine serum (FBS) (washing buffer). Bone marrow contents was centrifuged and the cell pellet was re-suspended and was layered over same volume of Ficoll-Paque (Innotrain, Germany) and centrifuged at 850×g for 25 minutes at 4°C. In the following, mononuclear cell layer was collected and was re-suspended in Dulbecco’s modified Eagle’s medium (DMEM) culture medium containing 10% FBS. Cell cultures were incubated in a 37°C incubator and passaged with 0.25% trypsin/ethylene diamine tetra acetic acid (EDTA).^19^ A general overview of methods steps was described as Figure 1.

### Characterization of BMSCs by cell surface markers detection and multi-lineage differentiation

Flow cytometry was used for immune-characterization of BMSCs as previously described by Fathi et al.^18^ Briefly, 10×10^4^ BMSCs were trypsinized and incubated with 5 µL of fluorescein isothiocyanate-conjugated antibody CD31 and CD34 and phycoerythrin–conjugated CD73 and CD44 (BD Pharmingen, USA) for 40 minutes on ice. At the end of incubating time, FACS instrument (Becton, USA) was used to quantify the fluorescence intensity of cells. In addition to flow-cytometry, the multipotency of BMSCs was also assessed. For this purpose, BMSCs were cultured in one of the adipogenic, osteogenic and chondrogenic induction medium as previously reported by Fathi and Farahzadi.^20^ At the end of 21th day, the BMSCs were fixed with 4% paraformaldehyde and were subjected to Sudan III (1% in 96% ethanol), Alizarin red (2% in distilled water) and toluidine blue (0.1% in distilled water) for adipogenesis, osteogenesis and chondrogenesis, respectively.^21^

### Culture of myeloid leukemia cell line K562

K562 (CML cell line) was purchased from Institute Pasteur (Iran) and was cultured in Roswell Park Memorial Institute (RPMI)-1640 supplemented with 10% (v/v) FBS.

### Co-culture of BMSCs and CML-cell line (K562)

BMSCs was plated into 6-well trans-well platesat 10×10^4^ cells/well in DMEM complete culture medium solution. After 24 h, 10×10^5^ K562 cells/well was added respectively into two groups; control group (culture of K562 alone) and experimental group (co-cultured K562 and BMSCs). After end of treatment time (7 days), two groups of cells were subjected to DNA isolation for measuring telomere length and protein extraction for western blotting and PCR-ELISA TRAP assay. Also, at the end of 7th day, culture media of two groups were collected for cytokine measuring by ELISA.

### Real-time PCR assessment

At the end of co-culture period, 10×10^5^ K562 cells/well from control group as well as experimental group were collected and total RNA from the cells was isolated and cDNA was synthesized using 2 µg RNA with YTA kit (Yekta Tajhiz Azma, Iran) as manufacturer’s instruction, respectively.^22^ The mRNA expressions of target genes in this experiment included *hTERT*, *P53*, *Wnt5a*, *β-catenin* and *β-actin*. Fluorescence data was analyzed to get CT values. The CT values were calculated in relation to *β-actin* CT values by the 2^-ΔΔCT^ method. The primer sequences are listed in Table 1.

**Table 1:** Primer sequences used for the real-time PCR assays

**No.**	**Gene**	**Primer pair sequence (5'-3')**	**Product length (bp)**
NM_001193376.1	*hTERT*	CAGCAAGTTTGGAAGAACCC GACATCCCTGCGTTCTTGG	98
NM_199173.5	*Wnt-5a*	GGCAGCGAGGTAGTGAAGA TCAGCCAACTCGTCACAGTC	131
NM_001330729.1	*β-catenin*	CATCTGACCAGCCGACACC CGAATCAATCCAACAGTAGCC	137
NM_001126118.1	*P53*	TCAGTCTACCTCCCGCCATAA AGTGGGGAACAAGAAGTGGAG	86
NM_001101.4	*β-actin*	AAACTGGAACGGTGAAGGTG TATAGAGAAGTGGGGTGGCT	174

### Telomere and single copy gene (SCG) standard curve

A standard curve is created by dilution of known quantities of TTAGGG repeated 14 times. Also as discussed and shown by Farahzadi et al.^23^ 36B4, was used as a control for determining genome copies per sample. A serial dilutions of TEL STD A (10^-1^ [1.18 ×10^8^] through to 10^-6^ [1.18 × 10^3^] dilution) as well as SCG STD A (10^-1^ through to 10^-6^ dilution) was performed for generating a standard curve.

### DNA isolation and telomere length measurement

Genomic DNA was isolated from two groups of cells as explained above and samples were dissolved in 30 μL final volume, 2 μL of these samples were used for DNA concentration measurement by NanoDrop Spectrophotometer. In the following, absolute telomere length measurement was done by real-time PCR as previously reported by Farahzadi et al.^23^ Data obtained from real-time PCR technique to measure absolute telomere length were analyzed as kb/reaction and genome copies/reaction for telomere and SCG, respectively, as described in detail previously by O’Callaghan & Fenech^24^ and Farahzadi et al.^23^

### Telomerase activity assay

PCR-ELISA TRAP assay was performed to determine the telomerase activity. For this purpose, total protein was extracted from control and experimental groups and the relative telomerase activity was assessed using the Telomerase PCR-ELISA kit (Roche Life Science, Germany). In brief, cell extracts were incubated with biotin-labeled primers at 25°C, and the telomeric repeats added onto the ends of the primers were amplified by PCR. The PCR products were added to digoxigenin-labeled detection specific probes and following were allowed to bind to a streptavidin-coated 96-well plate. Finally, the optical density of the blue color was measured at 450 nm by ELISA reader (Dynatech, USA).

### Western blotting analysis for Wnt-5a, β-catenin and P53 protein expression

To investigate the Wnt-5a/β-catenin and P53 signaling pathways involved in BMSCs effect on K562 cells, these protein expression was assessed by western blotting. For this stage, K562 cells protein in both groups (control and experimental) were extracted and 50 μg of each cell protein sample was electrophoresed on 12% polyacrylamide slab gels and transferred to poly vinylidene difluoride membrane. In the following, the membrane was incubated with primary antibodies of Wnt-5a, β-catenin and P53 (1:500, Santa Cruz Biotechnology, CA) and was incubated with goat anti-mouse secon­dary antibody (1:5000 Santa Cruz) for 60 minutes at 25°C. Also, β-actin was used as the internal control to normalize. Next, the protein bands detected with X-ray film. Protein bands intensity were measured and then calculated the ratio of target protein/β-actin and the obtained values were graphed.

### Cytokine measuring by ELISA

Culture media was collected from each group including, K562 cell line, BMSCs and co-culture of K562 cell line with BMSCs. ELISA was performed according to the manufacturer’s instructions (ExCell Biology, Shanghai, China; CINC-1 from R&D Systems). Briefly, a 96-well plate was coated with detection Reagent A and stored overnight at 4°C. Then, 50 μL of cell culture media from each groups and standard solution were added into the 96-well plate, which had been coated with human interleukin (IL)-6, IL-8 and transforming growth factor beta (TGF-β) antibodies, and detected via the ELISA sandwich technique. After terminating the reaction, the optical density at a wavelength of 450 nm in each well was determined using a spectrophotometer.

### Statistical analysis

The data were analyzed by one-way and two-way ANOVA followed by Dunnett’s post hoc test. Values were measured statistically significant at *P* < 0.05 by GraphPad Prism version 6.01. A comprehensive overview of methods that have been used in this paper was described as Figure 1.

## Results

### Culturing and characterization of BMSCs

**Figure 2:**
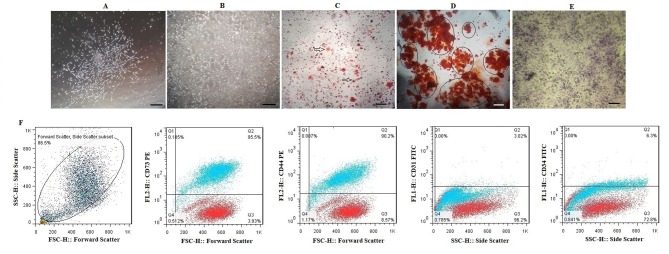
**Morphological features of bone marrow derived-MSCs;** (A) Spindle-shaped morphology of bone marrow cells at day 7, (B) More confluent bone marrow derived-MSCs at 21 days of culturing cells. **Multi-lineage differentiation of bone marrow derived-MSCs;**(C)Generation of lipid vacuoles after adipogenesis and staining by Sudan III; (D) Osteogenic differentiation and Alizarin red staining of mineralized cell aggregates; (E) Chondrogenic differentiation and Toluidine blue staining. **(F) The expression of the cell surface markers of bone marrow derived-MSCs that analyzed by flow cytometry.** Each antibody was tested individually and the isotopes controls were used as the negative control in this experiment; (A) a total population of cells, the bone marrow derived-MSCs were positive for CD73 (95.5%) and CD44 (90.2%) and negative for CD31 (3.02%) and CD34 (6.3 %). For CD31 and CD34, the isotype control was mouse IgG1 and for CD73 and CD44, the isotype control was mouse IgG2b. Also, isotype control is seen with red dots.

Bone marrow derived-MSCs like another MSCs, morphologically appear as spindle-shaped cells resembling fibroblasts (Figure 2A and B). After isolation and culturing of the BMSCs, adipogenesis and osteogenesis was done. Sudan III, Alizarin red and toluidine blue were used for staining the lipid droplets, mineralized compartments and aggrecan aggregates, respectively (Figure 2C-E). Immunophenotypic characterization of BMSCs was done by flow cytometry. Figure 2F shows that mesenchymal markers CD73 (95.5%) and CD44 (90.2%) are expressed. However, the hematopoietic markers CD31 (3.02%) and CD34 (6.3%) are not expressed in BMSCs.

### Investigation of telomere length, telomerase activity and hTERT gene expression

**Figure 3:**
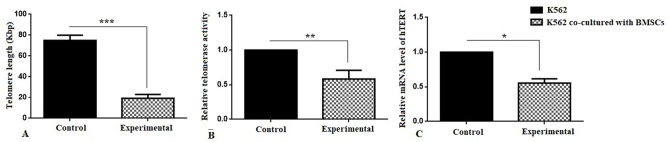
**(A) Absolute telomere length measurement;**BMSCs were significantly decreased the telomere length (***P <0.001) of K562 cell line compared with the control group. **(B) Relative telomerase activity measurement and (C) Relative*****hTERT*** **gene expression levels of K562 cell line at the end of 7th day co-culture period with BMSCs.**BMSCs were significantly decreased the telomerase activity (**P <0.01) and *hTERT* gene expression of K562 cell line (*P <0.05) compared with the control group.

Absolute telomere length measurement was evaluated at the end of 7th day co-culture period. As shown in Figure 3A, telomere length significantly decreased (19.13 kbp) compared to the control group (74 kbp) (***P < 0.001). Also, the telomerase activity has decreased by 50% in the experimental group compared to the control group (Figure 3B) (**P < 0.01). In addition, it was shown that the expression of *hTERT* gene was 0.4-fold decreased in the experimental group in compared with control group (Figure 3C) (*P < 0.05).

### BMSCs cause to change the gene and protein expression of Wnt-5a, β-catenin and P53 in K562 cell line

**Figure 4:**
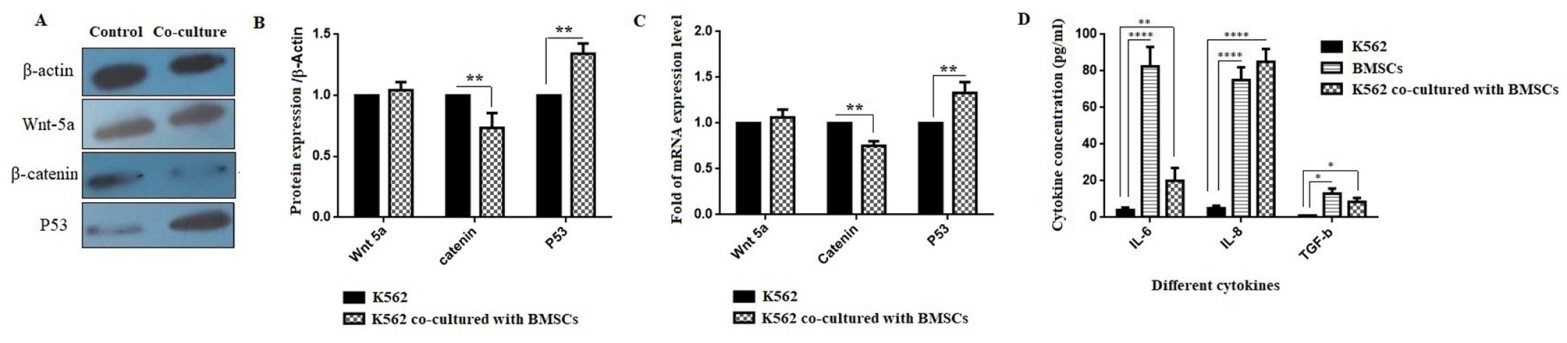
**Effect of bone marrow derived-MSCs on gene and protein expression of Wnt5a,β-catenin and P53 in K562 cell line.** (A and B) The protein and (C) the mRNA expression levels of Wnt5a, β-catenin and P53. (D) The secretion levels of cytokines TNF-α, IL-6, IL-8 and TGF-β (compared with control group) (*P < 0.05; **P < 0.01 and ***P < 0.001).

For evaluating the signaling pathways involved in the effect of factors and agents secreted from BMSCs on K562 cell, the protein and mRNA expression was examined by western blotting and real-time PCR, respectively. In this panel, the gene and protein expression of Wnt-5a, β-catenin and P53was investigated. As shown in Figures 4A and B, the protein expression levels of β-catenin and P53 were significantly decreased and increased, respectively (**P < 0.01). A significant decrease and increase in mRNA expression levels of β-catenin and P53, respectively, was also seen (Figure 4C) (**P < 0.01).

### Measuring cytokine secretion of BMSCs by ELISA

ELISA results revealed that the secretion of IL-6, IL-8, and TGF-β was significantly increased in both BMSCs and the co-culture of the K562 cell line with BMSCs groups compared to the K562 group (Figure 4D) (**P < 0.01 and ****P < 0.0001). In addition to IL-6 and IL-8, as shown in Figure 4D, the TGF-β level was markedly increased in both the BMSCs group and the co-cultured group compared with the K562 group (*P < 0.05).

## Discussion

This study demonstrated the effect of BMSCs on the K562 cell as a CML cell line by investigating telomere length, telomerase activity, and *hTERT* gene expression through P53, Wnt5a and β-catenin signaling pathways. To investigate the hypothesis of this research, K562 cells were co-cultured with BMSCs on trans-well plates for 7 days. To explore the mechanism of the effect of BMSCs on the CML cell line K562, at the end of co-culture period, K562 cells were collected and subjected to PCR-ELISA TRAP assay, quantitative real-time PCR, and western blotting to assay telomerase activity, telomere length, and protein expression, respectively. Also, the culture media was collected for measuring IL-6, IL-8, and TGF-β cytokines.

As seen from the results, *hTERT* gene expression, telomerase activity, and telomere length were significantly decreased in the experimental group (co-cultured K562 and BMSCs) by more than 3.9-, 0.5-, and 1.7-fold, respectively, when compared with the control group (culture of K562 alone).

Beside the studies on cancer, the potential role of telomerase upregulation in cancer and its inhibition in the cancer treatment were also of great interest and there are still many studies on this field. But the molecular mechanisms and signaling pathways involved are yet to be completely known and it seems at least some of the effects may be due to the same properties.^25^

Keller et al have previously shown that telomere length measured in peripheral blood of patients with CML correlates with disease stage, clinical prognostic scores, and response to treatment.^26^ Also, Samassekou et al reported that in the early stage of CML ontogenesis, long telomeres on key chromosomes may contribute to a cell proliferation advantage.^27^ In another study, Braig et al demonstrated that telomerase-targeting strategy could alleviate the tumor promoting effect of BCR-ABL via induce senescence in CML-like cells.^28^

The current results showed a significant relationship among *hTERT* gene expression, telomerase activity, and telomere length in K562 cells co-cultured with BMSCs. Since telomerase activity is regulated by the expression of the *hTERT* gene,^29^ a correlation between the telomerase activity and the expression of hTERT can be expressed.

Given the several involved mechanisms in telomerase activity, the regulation of telomere length seems to be complex. Based on the results of previous studies, it was hypothesized that the reductions in telomere length, telomerase activity and *hTERT* gene expression were heavily governed by cytokines.^30^ Among these factors, IL-6, IL-8, and TGF-β are possible candidates. In this study, cytokines were measured using ELISA.Results from ELISA demonstrated that the secretion of IL-6, IL-8, and TGF-β was significantly increased in both the control and the experimental groups compared with the K562 group. Liu and Hwang had measured cytokine expression by UCB-MSCs found that IL-6 and IL-8 were numerous proteins expressed, which agrees with our data.^31^

Two prominent cytokines (IL-6 and IL-8) detected in the co-culture were most probably derived from BMSCs, as the BMSCs culture alone indicated a high secretion of these cytokines, but they were absent in the K562 cell culture (no data obtained). Compared with the K562 cell culture, BMSCs secreted a great amount of both IL-6 and IL-8.

As previously shown by Klassen et al, IL-6 has wide range of performance for the differentiation, development, regeneration and tissue remodeling.^32,33^ The findings further showed that BMSCs co-cultured with K562 cells expressed higher amounts of IL-8 compared with the BMSCs cultured alone, consistent with previous reports.^34,35^

IL-8 as a pro-inflammatory cytokine has mitogenic and angiogenic potential. In the other words, IL-8 has been attracted as a main cytokine in the tumor progression in the modulation of angiogenesis.^36^ The reason for high expression of IL-6 and IL-8 in BMSCs cultured media remains to be investigated. In addition, TGF-β as an anti-inflammatory cytokine has apoptotic function to restrain cell proliferation and the loss of these effects leads to hyper proliferative disorders that are the hallmarks of tumors.^37^

Previous results of cytokine measurement showed detectable levels of TGF-β in the BMSCs and co-cultured media. It can be concluded that the presence of TGF-β is one reason for the decreasing telomere length and telomerase activity. These results confirm the results obtained in the current study.

As previously reported, MSCs are capable to inhibit the proliferation of cancer cells. In one study, Tyndall et al reported that MSCs being able to suppress immune reactions in an MHC-independent manner via their secretive soluble factors.^30^ In another study by Fonseka et al, it was demonstrated that MSCs derived from umbilical cord blood (UCB-MSCs) cause to significantly inhibited the proliferation of leukemic cells.^35^ Considering that the inhibition of tumor cells growth is dose-dependent, suggesting that the UCB-MSCs as well as other sources of MSCs such as bone marrow, adipose tissue etc can be used as anti-tumor agent.^38^ As indicated *in vivo* and *in vitro* study by Ahn et al, it was shown that the growth of tumor in mice was inhibited by adipose tissue-derived MSCs. Also, it was administered that homing of MSCs to tumor is well-established. It was shown that homing potential of MSCs can be mediated by cytokines secreted by tumors or/and their related stroma.^4^ These results confirm the results obtained by our study.

In addition to the mentioned items, many signaling pathways involved in the pathogenesis of carcinomas have been characterized; among these, the Wnt-5a/β-catenin and P53 pathways are important. During the activation process of the Wnt-5a/β-catenin signaling pathway and after entering β-catenin into the nucleus, the transcription of target genes including c-myc and cyclin D1 are activated. Improper activation of this pathway contributes to carcinogenesis and malignant behaviors.

In addition, the Wnt-5a/β-catenin pathway has been extensively investigated as a key target in cancer treatment and in fine-tuning the regulation of hematopoiesis.^39,40^ Previous studies were indicated that aberrantly expression and high expression of β-catenin cause to AML and poor prognosis in AML patients, respectively.^41,42^ Also it was reported that the Wnt/β-catenin pathway activation was detected in samples from patients with CML in blastic crisis. Additionally, it can be said that this study was confirmed the results from our study.

As mentioned above, the results of real-time PCR and western blotting showed that BMSCs could decrease the gene and protein expression of β-catenin. With all these interpretations, the molecular link between Wnt/β-catenin signaling and the expression of telomerase expression and telomere length was also reported by Hoffmeyer et al.^43^ It was shown that β-catenin-deficient mouse embryonic stem cells have short telomeres; conversely, stem cells expressing an activated form of β-catenin have long telomeres.

The direct relationship between the reduction in telomere length, telomerase activity and β-catenin expression was shown in this study. In addition to the Wnt/β-catenin pathway, the P53 pathway also plays an important role in cancer treatment targeting.^44^ In the present study, the mRNA and protein expression of p53 in K562 cells was significantly upregulated.

In one study, Basu and Haldar indicated that overexpression of P53 in some cancer cells via reducing the bcl-2 protein expression leading to in apoptotic cell death.^45^ In another study, Brassat et al reported that telomerase inhibition in CML patient is highly dependent on the presence of functional p53.^46^ These results are consistent with the results of the current study that overexpression of P53 is associated with decreasing telomeric length and telomerase activity. It can be concluded that the reduction in telomere length, telomerase activity and *hTERT* gene expression was governed by the P53, Wnt5a, and β-catenin signaling pathways.

## Conclusion

In conclusion, we provided evidence showing that BMSCs has an effect on reducing the *hTERT* gene expression, telomerase activity and telomere length of K562 cell, which is brought about via P53, Wnt5a and β-catenin signaling pathways. Without any ethical concerns, MSCs are easily obtained and cell therapeutic strategy using these cells seems to be a better choice for cancers, however, further researches are needed to use MSCs as clinical application.

## Ethical Issues

This study was approved by ethical committee of Tabriz University of Medical Sciences, Tabriz, Iran (Ethical No: IR.TBZMED.REC.1396.849).

## Conflict of Interest

The authors declare that they have no conflict of interest.

## Funding

This study was supported by a grant of Research Vice-Chancellor of Tabriz University of Medical Sciences, Tabriz, Iran.
